# On Pomade and Solution of Iodol

**Published:** 1886-10

**Authors:** 


On Pomade and Solution of Iodol.—Iodol, discovered by Messrs. Slider and Ciamicios, has been tried at the surgical clinic in Rome by Dr. Gaetino Maezonni. This product represents an antiseptic very much superior to iodoform, of which it does not share the disagreeable odor, nor the toxic properties. In addition to its antiseptic properties, iodol anæsthetizes the point of application and facilitates cicatrization. The remedy comes in the shape of a fine powder, and is used in the shape of a pomade or alcoholic solution as follows:

SOLUTION.

℞ Iodol, - - - - - - gr. L.
Alcohol, - - - - - - ℥ i
Glycerine, - - - - - - ℥ ii M.

POMADE.

℞ Iodol, - - - - - - ʒ i.

Vasaline, - - - - - - ʒ i. M.

—*Med. Age.*

